# Postsynaptic density levels of the NMDA receptor NR1 subunit and PSD-95 protein in prefrontal cortex from people with schizophrenia

**DOI:** 10.1038/npjschz.2015.37

**Published:** 2015-10-28

**Authors:** Vibeke Sørensen Catts, Dominique Suzanne Derminio, Chang-Gyu Hahn, Cynthia Shannon Weickert

**Affiliations:** 1 Schizophrenia Research Institute, Sydney, NSW, Australia; 2 Schizophrenia Research Laboratory, Neuroscience Research Australia, Sydney, NSW, Australia; 3 School of Psychiatry, University of New South Wales, Sydney, NSW, Australia; 4 Department of Psychiatry, University of Pennsylvania, Philadelphia, PA, USA

## Abstract

**Background:**

There is converging evidence of involvement of *N*-methyl-d-aspartate (NMDA) receptor hypofunction in the pathophysiology of schizophrenia. Our group recently identified a decrease in total NR1 mRNA and protein expression in the dorsolateral prefrontal cortex in a case-control study of individuals with schizophrenia (*n*=37/group). The NR1 subunit is critical to NMDA receptor function at the postsynaptic density, a cellular structure rich in the scaffolding protein, PSD-95. The extent to which the NMDA receptor NR1 subunit is altered at the site of action, in the postsynaptic density, is not clear.

**Aims:**

To extend our previous results by measuring levels of NR1 and PSD-95 protein in postsynaptic density-enriched fractions of prefrontal cortex from the same individuals in the case-control study noted above.

**Methods:**

Postsynaptic density-enriched fractions were isolated from fresh-frozen prefrontal cortex (BA10) and subjected to western blot analysis for NR1 and PSD-95.

**Results:**

We found a 20% decrease in NR1 protein (*t*(66)=−2.874, *P*=0.006) and a 30% decrease in PSD-95 protein (*t*(63)=−2.668, *P*=0.010) in postsynaptic density-enriched fractions from individuals with schizophrenia relative to unaffected controls.

**Conclusions:**

Individuals with schizophrenia have less NR1 protein, and therefore potentially fewer functional NMDA receptors, at the postsynaptic density. The associated decrease in PSD-95 protein at the postsynaptic density suggests that not only are glutamate receptors compromised in individuals with schizophrenia, but the overall spine architecture and downstream signaling supported by PSD-95 may also be deficient.

## Introduction

There is converging evidence of involvement of* N*-methyl-d-aspartate (NMDA) receptor hypofunction in the pathophysiology of schizophrenia. The earliest clue came from the observation that noncompetitive antagonists of the NMDA receptor, phencyclidine, and ketamine, produce behavior indistinguishable from the symptoms and cognitive deficits of schizophrenia (for review see ref. 1). More recently, NMDA receptor encephalitis,^2^ where individuals with high cerebrospinal fluid titers of autoantibodies against NMDA receptor subunits develop psychosis possibly due to antibody-mediated receptor endocytosis in brain,^3^ has also mechanistically implicated the NMDA receptor in schizophrenia. Studies in postmortem prefrontal cortex from individuals with schizophrenia have revealed no statistically significant change in NMDA receptor binding of ligands specific for the intrachannel PCP site.^4–10^ However, access of the NMDA receptor ligands to the PCP site is dependent on the NMDA receptor channel being open and accessible and may be expected to be quite variable from person to person. Other studies have utilized ligands for the NR2 glutamate site^4^ the NR2 polyamine binding site^4,8^ or the NR1 glycine site,^4,11,12^ but again results for prefrontal cortex have not found a change in binding in schizophrenia. In contrast, some postmortem studies of the expression of the obligatory NMDA receptor subunit, NR1, have observed decreased protein levels in telencephalon from people with schizophrenia,^13,14^ whereas other studies have found no change.^15–20^ The decrease in NR1 protein we observed in our study^14^ equates to an effect size of 0.53, indicating that 35 individuals per group are required to achieve 70% power to detect statistically significant changes in NR1 protein expression. Thus the majority of postmortem studies that find no statistically significant changes in NR1 protein levels or NMDA receptor ligand binding may not have been adequately powered to do so, contributing to conflicting results. We hypothesize that the decrease in NMDA receptors in schizophrenia can be detected with adequate sample size and with more anatomical precision.

Genetic and neuropathological findings in schizophrenia converge on differences in synaptic signaling,^21,22^ which is the primary function of the NMDA receptor. The postsynaptic density (PSD) is rich in PDZ (PSD-95, Dlg (discs large homolog), and ZO-1 (zona occludens)) domain-containing proteins, including the membrane-associated guanylate kinases (MAGUKs). These proteins are the scaffolding for the PSD, facilitating linkage between signaling molecules and to the actin skeleton.^23^ One of the most important MAGUKs is DLG-4, more commonly known, and herein referred to, as PSD-95. PSD-95 is especially important for the PSD structure, with the number of PSD-95 molecules determining the size of the PSD,^24^ and influencing the stabilization of the postsynaptic spine structure.^25^ Most molecular studies of PSD-95 protein levels in prefrontal cortex find no change in individuals with schizophrenia,^26–30^ though studies of the anterior cingulate cortex have observed a decrease in PSD-95 protein levels in individuals with schizophrenia.^26,27,30^ It is noteworthy that while reduced spine density in layer III of prefrontal cortex from people with schizophrenia has been reported,^31–33^ this reduction may pertain to primarily one cortical layer and to the subset of basilar dendrites.^34^ While a reduced spine density would suggest a reduction in PSDs from people with schizophrenia, this may not be evident in homogenate derived from all six cortical layers. Further to this, PSD-95 and other scaffolding proteins associate with receptors not only at the PSD, but also in the endoplasmic reticulum and Golgi network, where these proteins are processed and assembled before they are trafficked to the synapse. Thus, the total homogenate of grey matter includes pools of receptor proteins (i.e., NR1) and MAGUKs (i.e., PSD-95) in the endoplasmic reticulum and the Golgi network. The total homogenate of brain tissue as used in most published studies to date also includes receptor proteins within vesicles at the synapse, either recycling vesicles returning receptors to the cell membrane or endosomal vesicles targeting proteins for lysosomal degradation. The total pool of NMDA receptor subunits or scaffolding proteins may not accurately reflect the situation at the synapse, and a more informative determination of these proteins in individuals with schizophrenia may be measurements in PSD-enriched fractions.

Only two groups have measured NR1 and PSD-95 protein in synaptosomal extracts from people with schizophrenia.^28,35^ The first study used immunoprecipitation of PSD-95 from PSD-enriched fractions of prefrontal cortex followed by western blotting to detect increased amounts of NMDA receptor NR1 and NR2A subunits associated with PSD-95 in 10 elderly individuals with schizophrenia. The second study utilized mass spectrometry of pooled PSD-enriched samples (*n*=10/group) of anterior cingulate cortex and identified 143 differentially expressed proteins from people with schizophrenia compared with matched controls.^35^ Although levels of NMDA receptor subunits appeared not be to be significantly altered, the group did identify five differentially expressed proteins (4 decreased and 1 increased) that are known to interact with the NMDA receptor. The result of the first study seems contrary to our hypothesis; however, the second study does lend some support to changes in NMDA receptor partner proteins in synaptic spines in people with schizophrenia. Thus, further studies interrogating the status of synaptic NMDA receptor as well as PSD-95 in larger cohorts of people with schizophrenia are warranted.

Acknowledging the heterogeneity of research findings in the study of postmortem brain from individuals with schizophrenia, and recognizing that we observed a decrease in total NR1 protein overall, we tested if NR1 may be decreased rather than increased in PSD-enriched fractions derived from prefrontal postmortem tissue from the same brains we studied previously.^14^ To aid in the interpretation of our finding, we also measured levels of PSD-95 protein. We hypothesized that there would be a decrease in both NR1 and PSD-95 proteins in the PSD-enriched fractions from individuals with schizophrenia.

## Materials and methods

### Subjects

Postmortem tissue was obtained from 37 individuals with schizophrenia and 37 controls (demographic summary in Table 1) within the New South Wales Tissue Resource Centre collection. This study was carried out in accordance with the latest version of the Declaration of Helsinki after review by the Human Research Ethics Committee at the University of New South Wales (HREC No 07261 and 12435).

### Isolation of PSD-enriched fractions from human postmortem tissue

Approximately 400 mg (Table 1) of grey-matter tissue was sampled from the prefrontal cortex (BA10) situated in 1-cm thick coronal blocks snap-frozen following autopsy. The tissue was pulverized on dry ice and then homogenized in 5 ml buffer A (0.32 M sucrose, 1 mM NaHCO_3_, 1 mM MgCl_2_, 0.5 mM CaCl_2_) using a Potter–Elvehjem homogenizer to yield the ‘Total fraction’. This suspension was further processed using the protocol (Method 3) previously described in detail by Hahn *et al*,^36^ with modifications. The ‘Total fraction’ was centrifuged at 16,000*g* to yield the ‘Cytosolic fraction’ (supernatant). The pellet was resuspended in buffer B (0.32 M sucrose, 1 mM NaHCO_3_) which was overlaid onto a three layer sucrose gradient (1.2, 1, and 0.85 M sucrose) before being spun at 100,000*g* for 3 hours. The band at the interface between 1.2 and 1 M sucrose was collected and diluted with Solution B and centrifuged at 35,000*g*. The resulting pellet was resuspended in 0.1 mM CaCl_2_ and centrifuged at 15,000*g*. The pellet was solubilized in 20 mM Tris pH 7.4 with ‘triple detergent’ (final concentrations: 0.5% digitonin; 0.2% sodium cholate; 0.5% NP-40). An aliquot was set aside as the ‘Synaptic membrane fraction’, whereas the remainder was diluted to 1 ml with 0.1 mM CaCl_2_. One milliliter of 40 mM Tris-HCl pH 6 supplemented with 2% Triton-X 100 was added to the suspension and agitated at 4 °C for 30 min before being centrifuged at 35,000*g*. The supernatant was saved as the ‘Synaptic vesicle fraction’ and the pellet resuspended in Tris buffer at pH 8 supplemented with 2% Triton-X 100 and agitated at 4 °C for 60 min before being centrifuged at 140,000*g*. The supernatant was saved as the ‘presynaptic-enriched fraction’ and the pellet solubilised in 20 mM Tris pH 7.4 with ‘triple detergent’, yielding the ‘PSD-enriched fraction’. All buffers were supplemented with protease and phosphatase inhibitor cocktails (Sigma-Aldrich, Sydney, NSW, Australia). Protein concentrations were quantified using a bicinchoninic acid (BCA) based kit (ThermoFisher Scientific, Scoresby, VIC, Australia). The levels of NR1 and PSD-95 proteins in PSD-enriched fractions and of PSD-95 protein in the total fraction were quantitatively analyzed in the present study.

We commenced the tissue fractionation procedure with equal amounts of brain tissue from each diagnostic group (432±85 vs. 429±61 mg, *t*(71)−0.178, *P*=0.859). The yield of protein following homogenization of the tissue in sucrose buffer (Total fraction) did not differ between controls and individuals with schizophrenia (63.7±16.5 vs. 58.8±15.1 μg protein/mg tissue, t(71)−1.330, *P*=0.188). Similarly, the yield of protein in the PSD-enriched fraction did not differ between controls and individuals with schizophrenia (0.177±0.083 vs. 0.165±0.097 μg protein/mg tissue, *t*(66)−0.552, *P*=0.583).

### Immunoblotting

Western blotting was carried out using standard procedures as described previously.^14^ The amounts of protein loaded for each protein of interest were determined by running standard curves of protein (NR1 range 0.25–1.5 μg; PSD-95, T fraction range 2–8 μg; PSD-95, PSD-enriched fraction range 0.5–3 μg), and the optimal amount of protein was chosen to be within the linear range of the optical densities measured (NR1 1 μg; PSD-95 in T fraction 5 μg; PSD-95 in PSD-enriched fraction 1.5 μg). Protein was diluted in Laemmli buffer and separated by gel electrophoresis on 8% Bis/Tris acrylamide gels. Proteins were transferred onto nitrocellulose membranes, which were then blocked in 5% skim milk in Tris-buffered saline with 0.1% Tween-20 (TBST). Membranes were incubated with primary antibodies at 4 °C overnight (see Table 2 for details), then washed in TBST and incubated with species appropriate peroxidase-conjugated affinity-purified secondary antibodies at room temperature for 1 h. After further washing in TBST, membranes were incubated with chemiluminescence reagent (Merck Millipore, Sydney, NSW, Australia) and the band intensity was either captured and adjusted to the signal was not at saturation by Chemidoc (NR1) or the band intensity was determined from examining several exposure times to autoradiographic film and choosing the optimal exposure (PSD-95). Using these images, we determined levels of protein in bands of interest by densitometry, choosing a box size to encompass the largest band, and keeping the box size constant for each protein of interest across all samples within each experimental run. The laboratory researchers (DSD and DB) were blinded to the group allocation up until this point, when they were unblinded. For each occasion, NR1 and PSD-95 protein levels were normalized to the mean of control samples on each individual gel and to levels of β-III-tubulin or actin, respectively, both of which have previously been found to be unaltered in brains of individuals with schizophrenia.^37^ Each PSD-enriched fraction sample was analyzed on three separate occasions for NR1 analysis, and twice for PSD-95 analysis. The average value for each sample from all of the multiple replicates were utilized in data analysis and preparation of the figures. Average levels of β-III-tubulin and actin in PSD-enriched fractions did not differ significantly between individuals with schizophrenia and controls (Supplementary Figure 1). PSD-95 protein in the total homogenate was analyzed by western blot from one assay.

**Figure 1:**
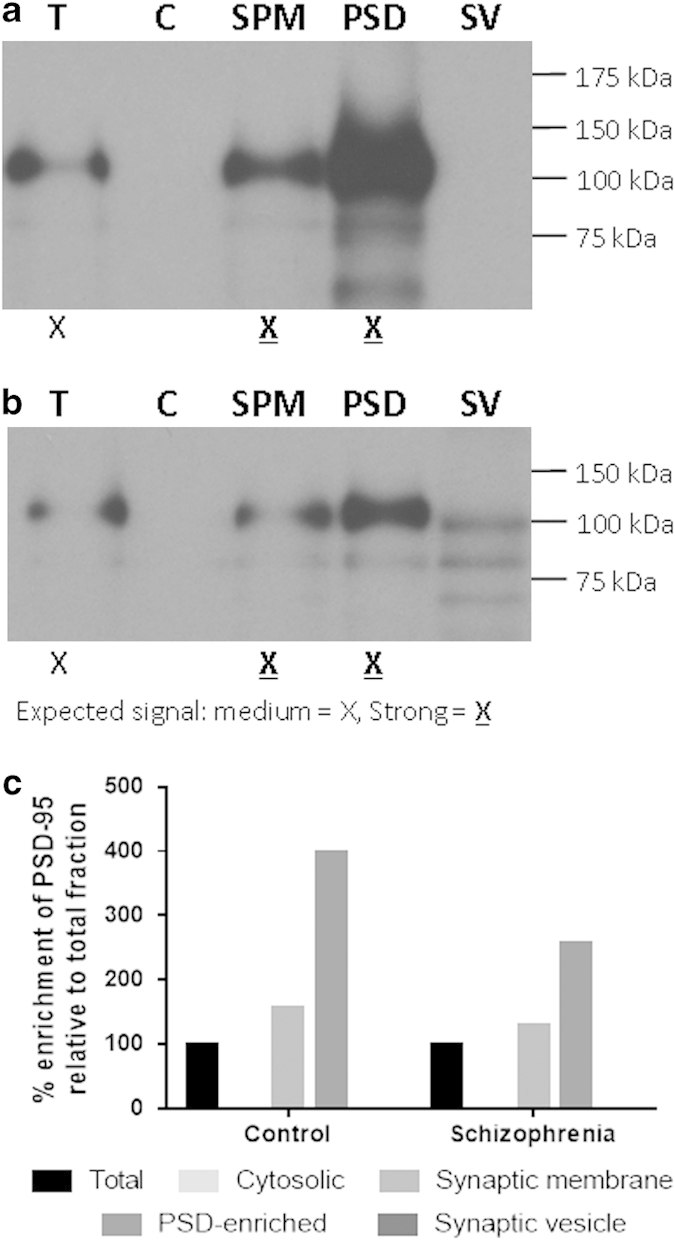
PSD-95 in tissue fractions from prefrontal cortex. Successful enrichment of the postsynaptic density (PSD) is indicated by the presence of increasing concentrations of PSD-95 in 1.5 μg of ‘total fraction’ (T), ‘synaptic membrane fraction’ (SPM) and ‘PSD-enriched fraction’ pooled samples (**a** and **b**). Quantification of band densities suggests greater enrichment of PSD-95 in control than in schizophrenia PSD-enriched samples (**c**). C, cytosolic fraction; SV, synaptic vesicle fraction.

**Figure 2:**
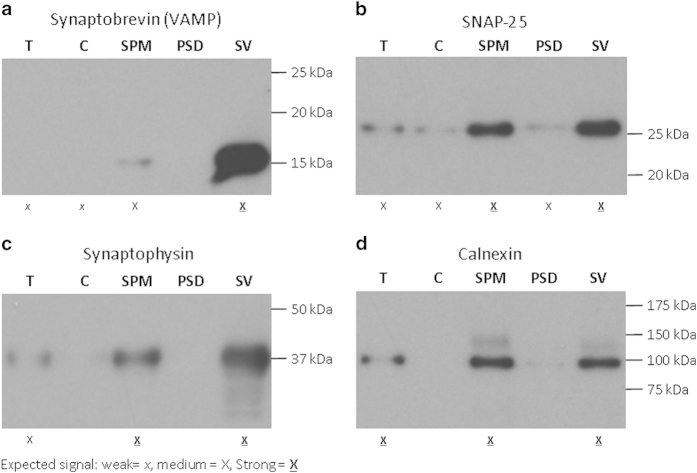
Presynaptic, vesicle and endoplasmic reticulum protein in tissue fractions from prefrontal cortex. The absence of α-synaptophysin, SNAP-25 and synaptobrevin (presynaptic vesicle proteins) in the PSD-enriched fraction (PSD) and their presence in the synaptic vesicle (SV) fraction attests to the success of the tissue fractionation procedure (**a**–**c**). The absence of calnexin, an endoplasmic reticulum marker in the PSD-enriched fraction indicates there is no significant contamination of PSD-enriched fraction with organelle membranes (**d**). C, cytosolic fraction; T, total fraction; SPM, synaptic membrane fraction.

Fidelity of the tissue fractionation procedure was confirmed by western blotting. Five control subjects were chosen at random to create a pooled protein samples (each subject contributing equal amounts of protein to the pooled samples). Similarly, pooled protein samples were created using fractions from two schizophrenia subjects. We loaded 1.5 μg protein for the two sets of five fractions obtained (Figures 1 and 2). The membranes were probed with primary antibodies for SNAP-25 (1:2,500 dilution, NCL-SNAP-25, Novocastra, Leica Biosystems, Noth Ryde, NSW, Australia), synaptobrevin (1:3,000 dilution, MAB 373, Chemicon, Merck Millipore, Sydney, NSW, Australia), calnexin (1:1,000, Cat # 2433, Cell Signaling, Merck Millipore) PSD-95 (Table 2) and α-synaptophysin (1:5,000 dilution, MAB329, Merck Millipore) followed by horseradish peroxidase conjugated secondary antibody (1:5,000 dilution, anti-mouse AP124P or anti-rabbit AP188P as appropriate, Merck Millipore).

### Analysis

Statistical analyses were performed using SPSS (version 22, IBM, Armonk, NY, USA). NR1 protein data were non-normally distributed within both diagnostic groups, and were log_10_ transformed for statistical analysis. Population outliers were defined as those values that fell outside of two s.d. from the diagnostic group mean after transformation (*n*=4/group on average). Parametric and non-parametric tests were performed as appropriate to determine diagnostic group differences in protein levels, and to examine whether gender, hemisphere, smoking status, typical versus atypical antipsychotic treatment (schizophrenia samples only), and/or suicide (schizophrenia samples only) were related to normalized NR1 and PSD-95 protein levels. All *P*-values reported are unadjusted (‘two-tailed’). We used Pearson correlations to relate protein levels within each group with demographic (age, pH, postmortem interval [PMI]) and, tissue specific (freezer storage time, brain volume, brain weight, protein yields) variables. In the schizophrenia group, we used Spearman and Pearson correlations as appropriate to relate protein levels to clinical variables (age at onset of schizophrenia, duration of illness, average daily chlorpromazine equivalent dose, last recorded chlorpromazine equivalent dose, lifetime chlorpromazine equivalent dose).

## Results

### Enrichment of PSD-95 across fractions

Successful enrichment of the PSD is indicated by the presence of increasing concentrations of PSD-95 in the ‘total fraction’ (T), synaptic membrane fraction’ (S) and the ‘PSD-enriched fraction’ (PSD) for both control (Figure 1a) and schizophrenia (Figure 1b) fractions. The enrichment of PSD-95 in the PSD-enriched fractions was considerably greater in the pooled control samples (398%) than in the pooled schizophrenia samples (256%) as indicated in Figure 1c.

### Protein measures in PSD-enriched fractions

We observed single bands of the predicted sizes for NR1 (~120 kDa), PSD-95 (~105 kDa), β-III tubulin (50 kDa), and actin (42 kDa). When the density of the NR1 and PSD-95 bands were determined, this resulted in a median NR1/β-III-tubulin ratio of 0.8 (range 0.05–2.85) and a median PSD-95/actin ratio of 0.8 (range 0.08–1.95) for the PSD-enriched fractions.

**Figure 3:**
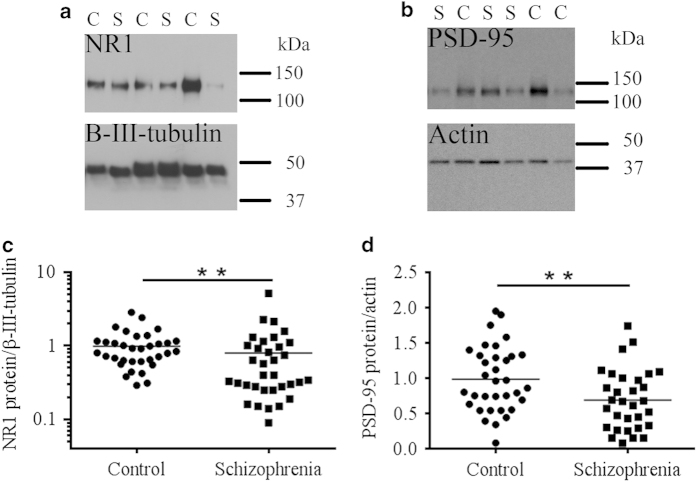
NR1 and PSD-95 proteins in PSD-enriched fractions from prefrontal cortex are reduced in people with schizophrenia. NR1 protein levels, normalized to β-III-tubulin, were quantified by western blot images captured by Chemidoc (representative blot captured on film shown in **a**). Single bands for NR1 and β-III-tubulin were present at the predicted sizes (~120 and 50 kDa, respectively). NR1 protein levels (average of three replicates) were decreased compared to matched controls (**c**). PSD-95 protein levels, normalized to actin, were quantified by western blot images captured on film (representative blot captured on film shown in **b**). Single bands for PSD-95 and actin were present in the predicted sizes (~105 and 42 kDa, respectively). PSD-95 protein levels (average of two replicates) were decreased compared with matched controls (**d**). **P<0.01; C, control; S, schizophrenia.

PSD-enriched fractions from people with schizophrenia had 20% less NR1 protein (t(66)=−2.874, *P*=0.006; Figures 3a and c) and 30% less PSD-95 protein (*t*(63)=−2.668, *P*=0.010; Figures 3b and d) than PSD-enriched fractions from controls. The levels of NR1 and PSD-95 protein in the PSD-enriched fractions correlated with each other (Pearson’s *r*=0.453, *P*<0.001, *n*=62). There was no statistically significant effect of diagnosis on the levels of PSD-95 protein in the total fraction (*t*(67)=−1.295, *P*=0.200). The amount of PSD-95 protein in the total fractions correlated significantly with that in the PSD-enriched fractions (*r*=0.257, *P*=0.045).

Demographic and clinical variables appeared to have little influence on levels of NR1 and PSD-95 protein measured in the PSD-enriched fractions or PSD-95 in the total fractions (Table 3). There were no significant correlations overall, or for each diagnostic group separately, for age at death. NR1 or PSD-95 protein levels did not differ significantly between males and females (NR1: (*t*(66)1.002, *P*=0.320); PSD-95 in PSD-enriched fraction: (*t*(63)0.259, *P*=0.797); PSD-95 in total fraction (*t*(67)0.471, *P*=0.639)), or between nonsmokers, smokers, and ex-smokers (NR1: (F(2,49)0.192, *P*=0.107); PSD-95 in PSD-enriched fraction: (F(2,50)0.659, *P*=0.522); PSD-95 in total fraction (F(2,52)1.152, *P*=0.324)). There were no significant correlations between PSD-95 or NR1 protein levels in PSD-enriched fractions or PSD-95 in total fractions, and duration of illness, age of onset of schizophrenia, last recorded or daily average or lifetime chlorpromazine equivalent doses (Table 3). Prescription of predominantly typical or atypical medication did not appear to have an effect on NR1 or PSD-95 (all ps>0.27) protein levels in the PSD-enriched fractions, or PSD-95 in the total fractions. In addition, whether a person with schizophrenia died following suicide or not did not appear to have an effect on NR1 or PSD-95 (all ps>0.245) protein levels in the PSD-enriched fractions.

Peri-mortem and technical factors had little appreciable effect on levels of NR1 and PSD-95 protein measured, with the exception of PMI, which correlated significantly with both NR1 and PSD-95 protein in the PSD-enriched fractions when the total group of samples was considered (Table 3), and with PSD-95 in the total fraction. However, when co-varying for PMI, the diagnostic differences in NR1 (F(1,65)=6.085, *P*=0.016) and PSD-95 (F(1,62)5.358, *P*=0.024) in the PSD-enriched fractions remained statistically significant, whereas PSD-95 in the total fractions (F(1,66)0.515, *P*=0.476) remained statistically non-significant. There were no significant correlations between NR1 and PSD-95 protein and tissue pH or between the amount of starting material, yield of protein in the total fraction or in the PSD-enriched fraction (Table 3). Gross morphological variables were not associated with NRI or PSD-95 protein levels, as there were no relationships with brain volume or brain weight (Table 3). There was no statistically significant effect of hemisphere on NR1 (*t*(66)-1.313, *P*=0.194) or PSD-95 (*t*(63)-1.946, *P*=0.056) protein in the PSD-enriched fractions nor on PSD-95 protein in the total fractions (*t*(67)-1.143, *P*=0.257).

## Discussion

We found decreased levels of the obligatory NMDA receptor NR1 subunit and of the PSD scaffolding protein, PSD-95 in PSD-enriched fractions from a large collection of prefrontal cortex (BA10) from individuals with schizophrenia and unaffected controls. The observed decrease in NR1 is consistent with our previous study of the neighboring DLPFC tissue, which demonstrated decreased levels of NR1 mRNA and protein in total homogenate from the same collection of individuals with schizophrenia studied here.^14^ It suggests that not only are the NR1 transcript and NR1 protein steady-state levels decreased in schizophrenia, but that this relates to less NR1 protein at the primary site of action, the PSD. This is important because studies of NR1 have suggested that this NMDA receptor subunit is normally produced in excess and is typically not a limiting factor in the number of NMDA receptor complexes being transported from the endoplasmic reticulum to the synapse.^38^ The overall reduction in NMDA receptor NR1 protein levels in total homogenate was ~36%, with a ~20% decrease in NR1 protein at the PSD in schizophrenia, suggesting these decreases are comparable and NR1-related deficits may occur at many biological steps. Indeed, previous work in a different tissue collection has suggested that trafficking of the NMDA receptor complex also is aberrant in individuals with schizophrenia.^26^

At face value our finding appears to be inconsistent with that reported by two previous studies.^28,35^ Hahn and colleagues found an increase in NR1 following immunoprecipitation of PSD-95 protein,^28^ whereas Focking and co-workers observed no change in NMDA receptor subunits.^35^ However, these studies are not directly comparable to ours. We measured total NR1 protein in the PSD-enriched fractions whereas Hahn and colleagues restricted their measure to NR1 and NR2A protein that was complexed with PSD-95. This suggests that there may be an increased interaction between NMDA receptor subunits and PSD-95, possibly indicating dysregulation of the protein interactions in the PSD-enriched fractions in brain from individuals with schizophrenia. Focking and co-workers defined their significantly changed proteins in their pooled PSD-enriched fractions as those which had a greater than 1.2-fold change by 2D gel electrophoresis. As our results suggest the group-wise decrease in NR1 in PSD-enriched fractions is 20%, a similar result in the Focking’s study might not have reached the threshold for significance. An alternative approach to determine the level of NMDA receptors at the synapse would be to measure levels of NR1 serine 897 phosphorylated protein, as this phosphorylation appears to be required for synaptic incorporation of the NMDA receptor.^39^ Western blot analysis by Emamian *et al*^15^ using a phosphor S897 NR1 specific antibody has previously revealed a significant decrease in phosphorylated NR1 protein in two collections of frontal cortex from individuals with schizophrenia. The latter study used two independent cohorts distinct from the one we studied, thus supports our current findings of reduced NMDA receptor at the synapse.

We also observed a decrease in PSD-95 protein in the PSD-enriched fraction in the absence of a change in PSD-95 protein in the total fraction. Previously we have measured PSD-95 mRNA in the DLPFC of the same tissue collection and found no change in transcript levels.^40^ These divergent results mirror the published literature, which mainly suggests no change or slightly decreased levels of molecular markers of excitatory synapses and spines, possibly due to disease heterogeneity or lack of anatomical resolution (reviewed in detail in ref. 41). Although morphological studies of Golgi-impregnated prefrontal cortex suggest a specific decrease in spine density on basilar dendrites in layer III, this appears not to be a pervasive deficit as this decrease does not appear to involve apical dendrites nor all neurons in all cortical layers.^31–34^ Consistent with this, we observed no statistical difference between schizophrenia and control samples in protein yield in the PSD-enriched fractions. Our observation of decreased PSD-95 would rather suggest smaller PSDs per spine, and perhaps more unstable spine structures, in individuals with schizophrenia relative to those in unaffected controls, as these two measures have been found to be related.^24,25^ Decreased PSD-95 at the synapse in turn may impair downstream signaling, and could be associated with alterations of the dendritic scaffolding proteins, microtubule-associated protein 2 (MAP2) and F-actin, in cortex from individuals with schizophrenia, as dendritic spine and MAP2 and F-actin pathologies appear to be intimately linked in temporal and dorsolateral prefrontal cortical regions in schizophrenia.^42,43^

Our study of NR1 in PSD-enriched fractions was strengthened by being carried out in the same collection of brain in which we had previously identified NR1 expression deficits at the mRNA and protein levels. However, the study was limited by the practical necessity to perform the tissue fractionation in frontal cortex (BA10) rather than dorsolateral prefrontal cortex (BA46), where our previous measures were obtained. The study was also limited by not expanding the panel of proteins investigated to the NR2 subunits and scaffolding proteins beyond the PSD-95. Future studies should include a broader spectrum of proteins at the PSD and not only the protein levels but also their phosphorylation states. Candidates for these studies could be identified using in silico gene prediction software applied to discovery data sets, such as that obtained by Focking and colleagues.^35^ A further limitation is our inability to determine whether the reduced PSD-95 and NR1 protein in the PSD-enriched fractions in individuals with schizophrenia is attributable to a trafficking deficit, whereby movement of these proteins from the endoplasmic reticulum to the postsynaptic density is retarded.^26^ Alternatively, as it would appear there is an altered composition of the postsynaptic density in schizophrenia, which may impact on its density and therefore its isolation by our gradient centrifugation protocol, it is also possible more of the postsynaptic density associated protein was lost or ended up in other fractions for the schizophrenia samples. Mechanistic insights might have been obtained by immunoprecipitation experiments along the lines of the Hahn’s study,^28^ which would also have allowed for direct comparison between our two studies. Although we were unable to identify any effects of antipsychotic medications on NR1 and PSD-95 levels at the postsynaptic density, this possibility will have to be considered in the interpretation of findings. In the case of NMDA receptor subunits, it would seem these are particularly decreased in antipsychotic-free individuals with schizophrenia in total homogenate^44^ and when indexed by *in vivo* binding to the intrachannel PCP-binding site of the SPET ligand CNS-1261.^45^ Further, NMDA receptor NR1 subunit levels in PSD were found to positively correlate with levels of antipsychotics, and analysis of rodents treated with antipsychotics suggest that these medications increase levels of NR1 protein in the PSD (Focking *et al.*, personal communication, and ref. 35). Taken together, these studies suggests that the findings of reduced NMDA receptor found in our study are not likely to be secondary to antipsychotic medication, and that had the individuals with schizophrenia in our study not been treated with antipsychotic drugs, their level of NR1 at the PSD might have been even lower.

In summary, our data on PSD-enriched fractions suggest that individuals with schizophrenia have less NR1 protein at the PSD, and therefore fewer functional NMDA receptors. As this is associated with decreased PSD-95 protein at the PSD, it suggests that not only is the detection of glutamate release compromised, but so may be downstream signaling.^46^ A final point of discussion is whether the NR1 and PSD-95 deficit primarily affects interneuronal or pyramidal cell glutamatergic synapses. The answer to this enquiry has important implications for the novel therapeutics targeting glutamatergic synapses, which are being trialed for the treatment of schizophrenia, as quite distinct strategies may be need to be developed to ameliorate a NMDA receptor deficit on inhibitory interneurons versus one on excitatory pyramidal cells. Unfortunately, the preparation of PSD-enriched fractions does not differentiate between synapses on interneurons and on pyramidal cells, but rather isolates the synapses based on their density. Future experiments should include measurement of cell-type specific expression levels of NR1 subunit, such as silver grain analysis of *in situ* hybridization for NR1. The design of experiments that delve deeper into subcellular localization and cell-type specificity of NMDA receptor deficits in schizophrenia are required to build the bridge between descriptive and mechanistic research necessary to test novel glutamate receptor based therapies.

## Figures and Tables

**Table 1:** Demographic, clinical and perimortem variables for postmortem brain collection

	*Control*	*Schizophrenia*	*Statistics*
*N*	37	37	
Age (years)	51.14±14.62	51.32±14.13	*t*(72) 0.057, *P*=0.955
Sex (male/female)	30/7	24/13	*χ* ^2^(1) 2.467, *P*=0.116
Hemisphere (left/right)	14/23	20/17	*χ* ^2^(1) 1.959, *P*=0.162
Postmortem delay (h)	24.80±10.97	28.46±13.77	*t*(72) 1.264, *P*=0.210
Tissue pH, cerebellum	6.52±0.31	6.44±0.26	*t*(72) −1.118, *P*=0.267
Freezer storage time (months)	69.6±42.7	79.9±37.2	*t*(72) 1.102, *P*=0.274
Brain volume (ml)	1438±122	1408±165	*t*(72) −0.893, *P*=0.375
Brain weight (g)	1446±127	1394±164	*t*(72) −1.525, *P*=0.132
Death by suicide (yes/no)	0/37	8/29	*χ* ^2^(1) 8.970, *P*=0.003
Smoking status (no/yes/ex-smoker)	13/9/7	7/23/0	*χ* ^2^(2) 14.912, *P*=0.001
Duration of illness (years)		27.62±13.82	
Age of onset (years)		23.70±6.07	
Medication (lifetime chlorpromazine)		7 907 586±7 953 627	

**Table 2:** Antibodies and consumables for western blotting

*Protein of interest*	*NR1*	*PSD-95*
Amount protein loaded	1 μg	5 μg for total fraction; 1.5 μg for PSD-enriched fraction
1° antibody for protein of interest	NR1; ~120 kDa; 1:1,000; ThermoFisher Scientific, SC-1467	PSD-95; ~105 kDa; 1:2,500; Antibodies Inc, #75-028
2° antibody for protein of interest	1:5,000, Merck Millipore AP192P donkey anti-mouse	1:2,500, Merck Millipore AP124P goat anti-mouse
1° antibody for housekeeping protein	Β-III-tubulin; ~50 kDa; 1:10,000; Sigma-Aldrich, T3952	Actin; ~42 kDa; 1:10,000; Merck Millipore, MAB1501
2° antibody for housekeeping protein	1:5,000 Merck Millipore AP188P mouse anti-rabbit	1:10,000 Merck Millipore AP124P goat anti-mouse
Image capture	Bio-Rad Chemidoc	Autoradiographic film
Densitometry software	Quantity One 1D Analysis Software v.4.6.5 (BioRad, Gladesville, NSW, Australia)	ImageJ

**Table 3:** Pearson (*r*) and Spearman’s (rho) correlation coefficients between PSD-95 and NR1 protein and demographic, clinical, perimortem, and technical variables

*Demographic variable*	*NR1 protein in PSD-enriched fractions*			*PSD-95 protein in PSD-enriched fractions*			*PSD-95 protein in total fractions*		
	*All subjects*	*Controls*	*Schizophrenia*	*All subjects*	*Controls*	*Schizophrenia*	*All subjects*	*Controls*	*Schizophrenia*
Age at death	*r*=−0.170	*r*=−0.192	*r*=−0.161	*r*=0.014	*r*=0.001	*r*=0.075	*r*=0.105	*r*=0.152	*r*=0.063
Postmortem delay (hours)	*r*=−0.246*	*r*=0.030	*r*=−0.274	*r*=−0.303*	*r*=−0.201	*r*=−0.329	*r*=−0.415**	*r*=−0.282	*r*=−0.464**
Tissue pH (cerebellum)	*r*=−0.086	*r*=−0.160	*r*=−0.131	*r*=−0.110	*r*=−0.285	*r*=0.097	*r*=−0.116	*r*=−0.121	*r*=−0.150
Freezer storage time (months)	*r*=0.150	*r*=0.021	*r*=0.308	*r*=0.042	*r*=0.217	*r*=−0.139	*r*=0.041	*r*=−0.071	*r*=0.174
Brain volume (ml)	*r*=−0.009	*r*=0.135	*r*=−0.145	*r*=0.026	*r*=−0.002	*r*=0.009	*r*=−0.074	*r*=−0.037	*r*=−0.106
Brain weight (gram)	*r*=0.044	*r*=0.191	*r*=−0.131	*r*=0.084	*r*=*r*=0.023	*r*=0.056	*r*=−0.090	*r*=−0.010	*r*=−0.170
Amount starting material (mg)	*r*=−0.090	*r*=−0.159	*r*=−0.054	*r*=−0.014	*r*=−0.011	*r*=−0.041	*r*=0.000	*r*=−0.163	*r*=0.162
Total fraction protein yield (mg)	*r*=0.105	*r*=0.109	*r*=0.030	*r*=−0.045	*r*=−0.086	*r*=−0.190	*r*=−0.025	*r*=−0.229	*r*=0.084
PSD-enriched fraction protein yield (μg)	*r*=0.151	*r*=0.066	*r*=0.162	*r*=0.130	*r*=0.120	*r*=0.124	*r*=−0.123	*r*=−0.164	*r*=−0.169
Duration of illness (years)			*r*=−0.148			*r*=0.127			*r*=0.087
Age of onset (years)			*r*=−0.035			*r*=−0.114			*r*=−0.051
Last recorded chlorpromazine equivalent dose			rho=0.022			rho=−0.055			rho=0.270
Average chlorpromazine equivalent dose			rho=−0.071			rho=−0.036			rho=0.257
Lifetime chlorpromazine equivalent dose			rho=−0.106			rho=0.049			rho=0.241

*P<0.05, **P<0.01.
