# Supplementary material for: Postsynaptic density levels of the NMDA receptor NR1 subunit and PSD-95 protein in prefrontal cortex from people with schizophrenia
Source: NPJ Schizophr. 2015 Oct 28;1:15037–. doi: 10.1038/npjschz.2015.37 (PMC4849460; doi:10.1038/npjschz.2015.37)
Supplement: Supplementary Figure 1 [file npjschz201537-s1.doc]

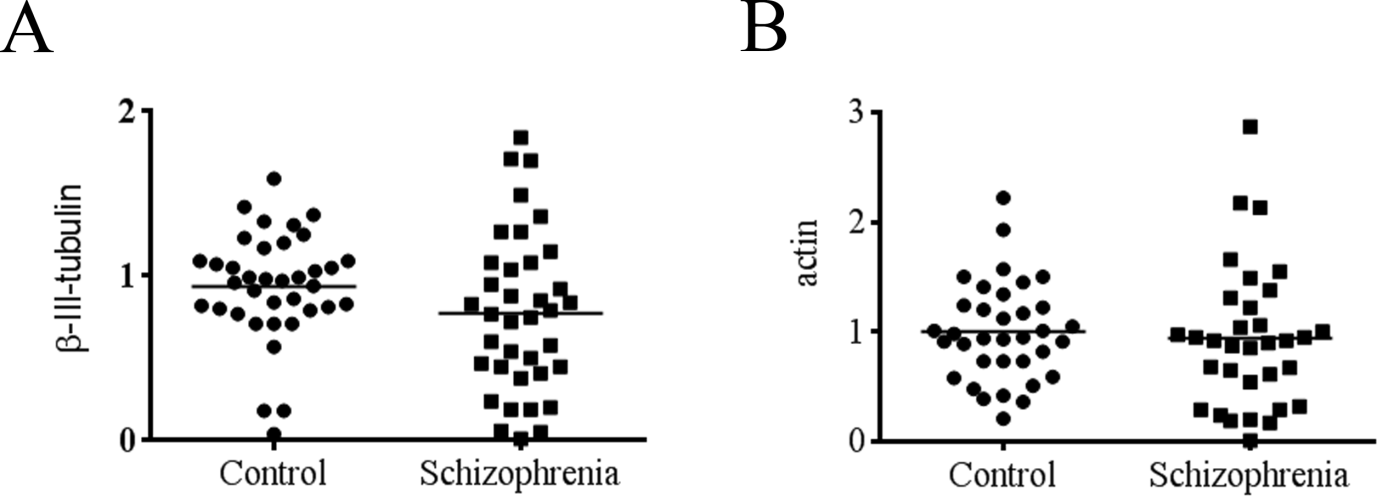


**Supplementary Figure 1.**

Β-III-tubulin and actin protein levels were quantified by Western blot. There were no statistically significant effect of diagnosis on β-III-tubulin levels (A), nor on actin levels (B).
